# False-positive *Neisseria gonorrhoeae* results on the Abbott Alinity m STI assay caused by *Neisseria meningitidis*: two cases from Australia

**DOI:** 10.1128/jcm.00347-26

**Published:** 2026-06-12

**Authors:** Janelle Maskell, Michael O'Connor, Monica M. Lahra, David M. Whiley, Sebastiaan van Hal, Todd M. Pryce

**Affiliations:** 1 Molecular Medicine, Royal Hobart Hospital 34379, Hobart, Tasmania, Australia; 2 Microbiology and Infectious Diseases, Royal Hobart Hospital 34379, Hobart, Tasmania, Australia; 3 World Health Organization Collaborating Centre for STI and AMR, New South Wales Health Pathology, Microbiology, The Prince of Wales Hospital, Randwick, New South Wales, Australia; 4 Faculty of Medicine, University of New South Wales 98994 , Sydney, New South Wales, Australia; 5 UQ Centre for Clinical Research, Faculty of Medicine, The University of Queensland, Brisbane, Queensland, Australia; 6 Pathology Queensland, Queensland Health, Brisbane, Queensland, Australia; 7 New South Wales Health Pathology, Microbiology, Royal Prince Alfred Hospital, Camperdown, New South Wales, Australia; 8 School of Medicine, University of Sydney 7799, Sydney, New South Wales, Australia; 9 PathWest Laboratory Medicine WA, Department of Clinical Microbiology, Fiona Stanley Hospital, Murdoch, Western Australia, Australia; Marquette University, Milwaukee, Wisconsin, USA

**Keywords:** Abbott Alinity m STI assay, *Neisseria meningitidis*, false-positive, *Neisseria gonorrhoeae*

## LETTER

We report two cases of *Neisseria meningitidis* (NM) isolated from pharyngeal swabs that yielded false-positive *Neisseria gonorrhoeae* (NG) results on the Abbott Alinity m STI assay (Alinity; Abbott Laboratories, Chicago, IL, USA), which includes a pharyngeal testing claim. These cases closely parallel the recent report from Sweden by Herrmann et al. (1), in which NM was misidentified as NG due to cross-reactivity with the Alinity *opa* gene target (1). Here, we describe similar findings from Tasmania, Australia. Both patients were male, asymptomatic, and underwent routine screening for sexually transmitted infections in June 2024 and October 2025. Pharyngeal swabs processed on Alinity returned NG-positive results with cycle of quantification (*C_q_*) values of 26.7 and 27.5, respectively. Confirmatory testing via the Xpert CT/NG assay (Xpert; Cepheid, Sunnyvale, CA, USA) was negative. Two NM isolates (NM1 and NM2) were recovered from culture and were identified by MALDI Biotyper (Bruker Daltonics, Billerica, MA, USA) with a high confidence score of >2.0.

Acknowledging that testing cultured isolates on commercial NAAT platforms falls outside manufacturers’ package insert claims, several colonies from each isolate were suspended in Alinity m multi-collect media and 1 mL of nuclease-free water for Alinity and Xpert testing, respectively. Alinity reproduced positive results from both isolates (*C_q_* < 15). Xpert testing was negative for the NG2 and NG4 targets. We also assessed potential cross-reactivity with other commercial screening and supplemental assays. Based on availability and timing, NM2 was referred to an external laboratory for additional testing using the cobas 6800 CT/NG assay (cobas CT/NG; Roche Molecular Systems Inc., Branchburg, NJ, USA), targeting DR-9, and the *ResistancePlus* GC assay (RP-GC; SpeeDx, Eveleigh, NSW, Australia), targeting *opa* and *porA* (2). Several colonies were suspended in cobas PCR media (Roche) and tested with both assays. The cobas CT/NG assay returned a negative result, while RP-GC was positive for *opa* (*C_q_* 17.5) and negative for *porA*. Whole-genome sequencing (WGS) confirmed that both isolates were capsular-null NM strains. NM1 was assigned to sequence type ST-11026 within clonal complex CC-32. Further genome analysis is underway to help clarify the cross-reactivity mechanism.

These findings reinforce the diagnostic challenge posed by genetic overlap between commensal and pathogenic *Neisseria* species, particularly in pharyngeal samples where NM colonization is common. Reliance on a single gene target, such as *opa*, may compromise specificity in low-prevalence settings like ours. Such false-positive results are concerning due to unnecessary antibiotic administration and potential adverse social and relationship consequences. Laboratories should be aware of this potential cross-reactivity, especially high-volume testing laboratories that use the Alinity system. Other high-throughput testing platforms that report performance claims for pharyngeal samples may also be at risk of reporting errors due to similar target recombination events. For example, we note a recent report of a commensal *Neisseria* species cross-reacting with the cobas CT/NG test (3). In conclusion, we support the recommendation by Herrmann et al. to incorporate multi-target confirmation and consider local epidemiology when interpreting NG results from pharyngeal sites. Collectively, these reports highlight the need for rigorous supplemental testing to address the ongoing issues of NG screening assay non-specificity (1–7).
